# New protocol for successful isolation and amplification of DNA from exiguous fractions of specimens: a tool to overcome the basic obstacle in molecular analyses of myxomycetes

**DOI:** 10.7717/peerj.8406

**Published:** 2020-01-24

**Authors:** Paulina Janik, Michał Ronikier, Anna Ronikier

**Affiliations:** W. Szafer Institute of Botany, Polish Academy of Sciences, Kraków, Poland

**Keywords:** Amoebozoa, Barcoding, Direct PCR, Herbarium collections, Non-destructive DNA sampling, Slime moulds, Type specimens

## Abstract

Herbarium collections provide an essential basis for a wide array of biological research and, with development of DNA-based methods, they have become an invaluable material for genetic analyses. Yet, the use of such material is hindered by technical limitations related to DNA degradation and to quantity of biological material. The latter is inherent for some biological groups, as best exemplified by myxomycetes which form minute sporophores. It is estimated that ca. two-thirds of myxomycete taxa are represented by extremely scanty material. As DNA isolation methods applied so far in myxomycete studies require destructive sampling of many sporophores, a large part of described diversity of the group remains unavailable for phylogenetic studies or barcoding. Here, we tested several procedures of DNA isolation and amplification to seek for an efficient and possibly non-destructive method of sampling. Tests were based on herbarium specimens of 19 species representing different taxonomic orders. We assayed several variants of isolation based on silica gel membrane columns, and a newly designed procedure using highly reduced amount of biological material (small portion of spores), based on fine disruption of spores and direct PCR. While the most frequently used column-based method led to PCR success in 89.5% of samples when a large amount of material was used, its performance dropped to 52% when based on single sporophores. Single sporophores provided amplicons in 89.5% of samples when using a kit dedicated to low-amount DNA samples. Our new procedure appeared the most effective (94.7%) while it used only a small fraction of spores, being nearly non-destructive; it was also the most cost-effective. We thus demonstrate that combination of adequate handling of spore micro-disruption coupled with application of direct PCR can be an efficient way to circumvent technical limitations for genetic studies in myxomycetes and thus can substantially improve taxon sampling for phylogeny and barcoding. Additionally, this approach gives a unique possibility to apply both molecular and morphological assays to the same structure (sporophore), which then can be further stored as documentation.

## Introduction

Herbarium collections (and biological museum collections in general) play an essential role as a source of biological material for documentation, monitoring and scientific purposes (Funk, 2003). Specimens and related data can be explored for a wide array of research including, for instance, taxonomical (e.g., Martin & Alexopoulos, 1969; Bebber et al., 2010; Petersen & Hughes, 2010), biogeographic (e.g., Wollan et al., 2008; Lavoie, 2013; Ronikier & Ronikier, 2010), global change (Meineke, Davis & Davies, 2018) and conservation biology studies (Greve et al., 2016). Over last years, with an increasing development of DNA-based molecular methods in ecology and evolution, herbarium specimens have appeared as an invaluable material for phylogenetic analyses (e.g., Moncalvo et al., 2002; Fiore-Donno et al., 2012; Fiore-Donno et al., 2013; Kistenich et al., 2019). In particular, type collections provide key reference for molecular barcoding databases used in biodiversity and environmental studies and provide irreplaceable source of specimens based on which the species’ name can be correctly applied (e.g., Larsson & Jacobsson, 2004; Lehtonen & Christenhusz, 2010; Chomicki & Renner, 2015; Dormontt et al., 2018).

Two main obstacles hinder the use of herbarium collections as a source for DNA isolation. One, affecting all types of collections, is DNA degradation mostly related to collection age, its preparation, treatment and storage conditions (Lehtonen & Christenhusz, 2010; Staats et al., 2011; Weißet al., 2016; Kistenich et al., 2019). The second limitation is quantity of available biological material, especially relevant in the case of organisms with small dimensions and those characterized by ephemeral emergence substantially limiting the possibility of sampling in the field. This latter limitation is inherent for myxomycetes and greatly influences the potential for phylogenetic and evolutionary studies in this group.

Myxomycetes (plasmodial slime moulds) are one of the largest groups of Amoebozoa (Adl et al., 2007; Pawlowski, 2014). They form a monophyletic group among amoebozoan protists (Kang et al., 2017) and belong to the least explored groups of soil protists (Urich et al., 2008; Geisen et al., 2015). A unique character that places them at an outstanding position among other amoebozoans is the presence of macroscopic sporophores produced during their life cycle. Due to the presence of these spore-bearing structures, myxomycetes have long been considered fungal-like organisms and, similar to fungi, they are routinely preserved in herbaria (Ronikier & Halamski, 2018). The great majority of species form small sporocarps of about 1–2 mm in diameter (Poulain, Meyer & Bozonnet, 2011). Sporocarps may be produced in large quantities, but more often one gathering consists of a small group of sporocarps or, when obtained using the moist chamber culture technique, only dormant stages or a few fruiting bodies may be produced (Stephenson & Stempen, 1994; Keller & Braun, 1999; Shchepin et al., 2017). Therefore, herbarium collections of myxomycetes usually contain small quantity of biological material. Since the first attempt to isolate DNA from herbarium specimens of myxomycetes (Martín, Lado & Johansen, 2003), there is an increasing interest to barcode myxomycete species and examine phylogenetic relationships between the taxonomic groups using molecular data and based on field-collected specimens deposited in herbarium collections (e.g., Fiore-Donno et al., 2005; Fiore-Donno et al., 2012; Fiore-Donno et al., 2013; Novozhilov et al., 2013b; Leontyev et al., 2014; Leontyev, Schnittler & Stephenson, 2015; Schnittler et al., 2017)). Almost all studies using molecular methods in myxomycetes are based on a large quantity of biological material derived from several up to 30 sporocarps per collection (see Table 1 for examples and references). There is no DNA isolation kit or method specifically dedicated to myxomycetes and several kits are in use. In most cases DNeasy Plant Mini Kit (Qiagen, Hilden, Germany), widely used also for DNA isolation from plants and fungi, is applied (Table 1). It requires destructive sampling of several to a few dozens of sporocarps. While there are attempts to find effective procedures to obtain good quality DNA for efficient downstream procedures from small quantities of biological material in various groups of organisms (e.g., Telle & Thines, 2008; Hofreiter, 2012; Shepherd, 2017; Cruaud et al., 2018), such endeavours were never focused on myxomycetes. Yet, available DNA isolation methods impose profound constraints for progress in myxomycete barcoding and phylogenetics. Schnittler & Mitchell (2000) estimated that 35% of myxomycete taxa described up till 2000 were known from their type locality only (one or a few collections) and further 30% remain rarely collected and reported (2–20 collections in total). Accordingly, it can be assumed that specimens of about two-thirds of all described taxa of myxomycetes are currently unavailable for molecular analyses because they are represented by too scanty material. It is therefore clear that the phylogeny of the whole group of myxomycetes cannot be solved using the methods used to date; this is valid by extension also for a taxonomically relevant barcoding reference database. An efficient methodological advancement is essential for overcoming these limitations. The aim of the present work is therefore to test selected approaches and propose a successful, possibly non-destructive protocol for isolation of DNA from small portions of herbarium specimens that could be universally applied to myxomycetes.

**Table 1:** DNA isolation procedures and amount of material used in published myxomycete studies.

	DNA isolation procedure/kit									
Source	Dneasy plant mini kit (Qiagen)	Invisorb Spin Food Kit II (Stratec)	E.Z.N. A. Plant DNA Kit (Omega Bio-Tec)	E.Z.N. A. Fungal DNA Kit (Omega Bio-Tec)	Invisorb Spin Plant Mini Kit (Stratec)	CTAB	AxyPrep Multisource Genomic DNA Miniprep Kit (Axygen)	PureLink Plant Total DNA Purification Kit (Thermo Fisher)	PreCellys 24 tube # VK05 (Bertin Technologies)	Amount of material
Martín, Lado & Johansen (2003)				+						not given
Fiore-Donno et al. (2005)	+									a few sporophores
Kamono & Fukui (2006)						+				not given
Fiore-Donno et al. (2008)	+									several sporophores
Kamono et al. (2009)						+				not given
Fiore-Donno et al. (2010)	+									several sporophores
Hoppe & Kutschera (2010)	+									0.05–0.1 g
Hoppe, Müller & Kutschera (2010)	+									0.05–0.1 g
Fiore-Donno et al. (2011)	+									5–6 sporophores
Fiore-Donno et al. (2012)	+									5–6 sporophores
Nandipati et al. (2012)									+	2–5 sporocarps
Erastova et al. (2013)	+									30 sporocarps
Fiore-Donno et al. (2013)	+									5–6 sporophores
Novozhilov et al. (2013a)							+			not given
Novozhilov et al. (2013b)	+									about 5 sporophores
Leontyev et al. (2014)		+								5–6 sporophores
Feng & Schnittler (2015)	+	+								5–20 sporophores
Leontyev, Schnittler & Stephenson (2015)		+			+					5–6 sporophores
Walker, Leontyev & Stephenson (2015)		+								5–6 sporophores
Wrigley de Basanta et al. (2015)	+									5–8 sporophores
Feng et al. (2016)		+								2–3 sporophores
Kretzschmar et al. (2016)	+									20–30 sporophores
Shchepin, Novozhilov & Schnittler (2016)								+		not given
Dagamac et al. (2017)			+							equivalence of 10–15 sporophores
Erastova, Novozhilov & Schnittler (2017)	+									6–10 sporophores
Feng & Schnittler (2017)	+	+								5–20 sporophores
Wrigley de Basanta et al. (2017)	+									5–8 sporophores
García-Martín, Mosquera & Lado (2018)	+									5–8 sporophores
Borg Dahl et al. (2018a)	+		+		+					3–6 sporophores
Borg Dahl et al. (2018b)			+							3–6 sporophores

## Material and Methods

### Biological material

In the design of sampling for testing different DNA extraction methods we assumed that efficiency and universality of the methods will be best assessed with a focus on a wide taxonomic sampling. We thus selected herbarium collections of 19 myxomycete species, representing different orders, and large enough for repeated removal of sporocarps (Table 2). All collections are deposited in the Collection of Myxomycetes of KRAM.

**Table 2:** Details of herbarium specimens used in the study and success (+) in amplification of partial SSU rDNA gene from myxomycetes collections using four tested procedures (see ‘Material and Methods’ for details).

						Approx. size of a single sporocarp			Procedures				
No.	Taxonomical group	Species	Specimen origin	Herbarium accession number at KRAM	Year of collection	Length (mm)	Width (mm)	Number of sporocarps used in ‘standard method’	1	2	3	4	GenBank accession number
1	Cri	*Cribraria* cf. *persoonii* Nann.-Bremek.	Poland	M-1075	2002	0.4	0.4	10	+	–	+	+	MN104036
2	Cri	*Licea parasitica* (Zukal) G.W. Martin	Poland	M-1599	2004	0.2	0.1	20	+	–	+	+	MN104046
3	Phy	*Diachea leucopodia* (Bull.) Rostaf.	Poland	M-1767	2000	0.8	0.5	6	+	–	+	+	MN104037
4	Phy	*Diderma microcarpum* Meyl.	France	M-1323	2007	0.6	0.6	7	+	–	+	+	MN104039
5	Phy	*Didymium nigripes* (Link) Fr.	Poland	M-1771	2003	0.5	0.5	8	–	–	–	+	MN104040
6	Phy	*Leocarpus fragilis* (Dicks.) Rostaf.	Poland	M-1769	2000	0.9	0.8	5	+	+	+	+	MN104044
7	Phy	*Lepidoderma chailletii* Rostaf.	Poland	M-1145	2004	0.8	1.0	5	+	+	+	+	MN104045
8	Phy	*Physarum andinum* A.Ronikier & Lado	Argentina	M-1541	2009	0.8	0.8	5	–	–	–	+	MN104050
9	Phy	*Physarum bivalve* Pers.	Poland	M-1768	2000	1.1	0.5	5	+	–	+	+	MN104051
10	Ste	*Comatricha nigra* (Pers. ex J.F. Gmel.) J. Schröt.	Finland	M-1766	1994	0.5	0.5	8	+	–	+	+	MN104035
11	Ste	*Diacheopsis metallica* Meyl.	France	M-1456	2008	1.1	1.1	5	+	+	+	+	MN104038
12	Ste	*Lamproderma cucumer* (Meyl.) Nowotny & H. Neubert	France	M-1338	2008	0.9	0.8	5	+	+	+	+	MN104042
13	Ste	*Lamproderma sauteri* Rostaf.	France	M-1334	2008	0.9	0.9	5	+	+	+	+	MN104043
14	Ste	*Stemonitopsis hyperopta* (Meyl.) Nann.-Bremek.	Poland	M-1087	2003	1.3	0.4	5	+	–	+	+	MN104052
15	Tri	*Arcyria obvelata* (Oeder) Onsberg	Poland	M-1083	2002	1.3	0.4	5	+	+	+	+	MN104034
16	Tri	*Hemitrichia minor* G. Lister	Portugal	M-1638	2015	0.5	0.5	8	+	+	+	+	MN104041
17	Tri	*Metatrichia floriformis* (Schwein.) Nann.-Bremek.	Poland	M-1770	2009	0.6	0.6	8	+	+	+	+	MN104047
18	Tri	*Oligonema schweinitzii* (Berk.) G.W. Martin	France	M-1466	2008	0.3	0.4	10	+	+	+	–	MN104048
19	Tri	*Perichaena corticalis* (Batsch) Rostaf.	Poland	M-1090	2003	0.3	0.4	10	+	+	+	+	MN104049

**Notes.** Taxonomical groups: Cri, Cribrariales; Phy, Physarales; Ste, Stemonitidales; Tri, Trichiales; (see Lado & Eliasson, 2017).

### DNA isolation methods

**Figure 1:**
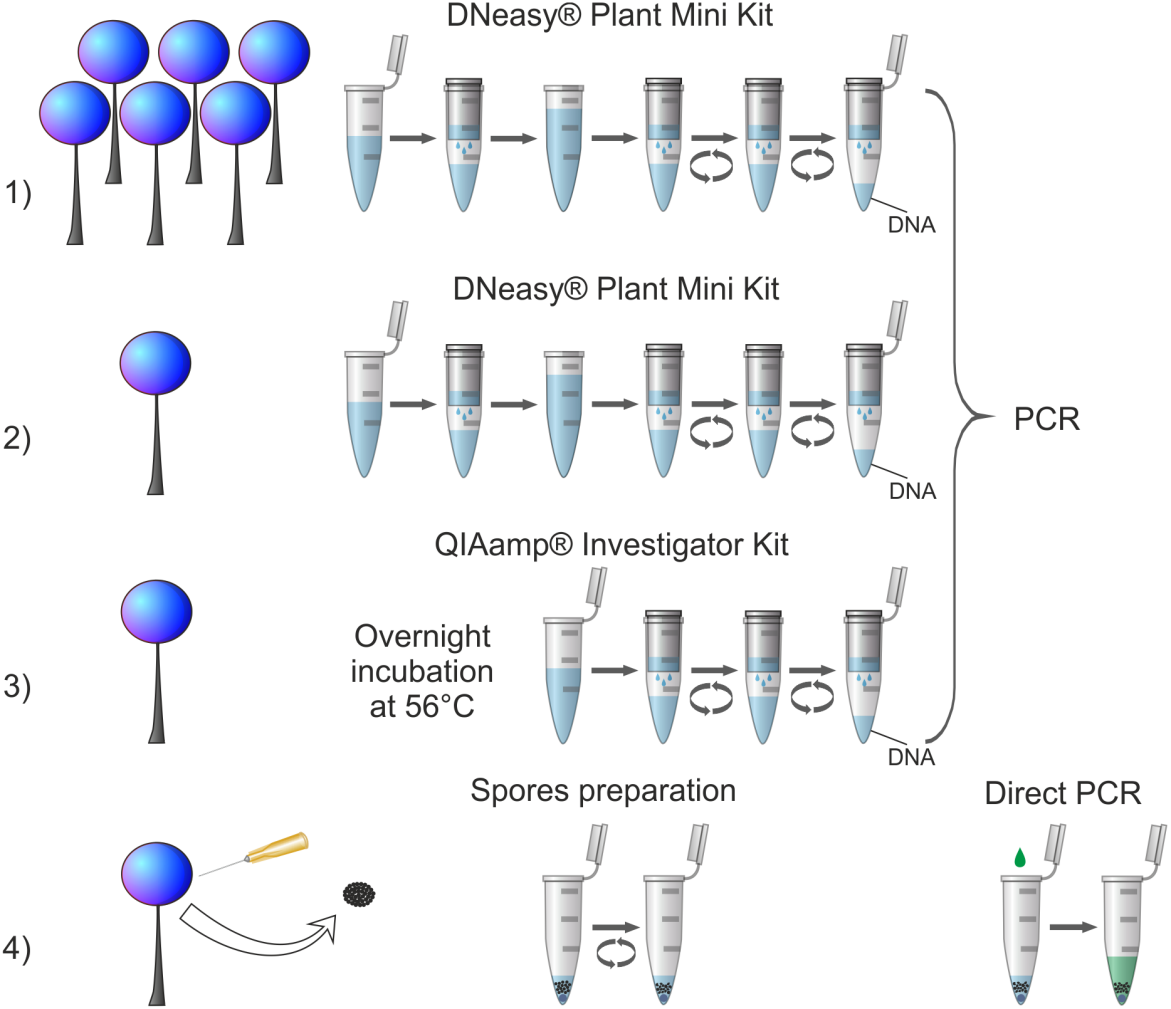
Comparison of applied DNA isolation/amplification procedures. Numbers indicate corresponding procedures described in the text. For procedures (1), (2) and (3) all steps of DNA isolation are visualised with respect to the manufacturer’s instructions; for all of them one standard PCR protocol was applied. For procedure (4) all applied steps are shown: intake of spores, spores disruption in a Tris-EDTA buffer by vortexing (spore preparation) and SSU rDNA amplification using direct PCR.

Four procedures of DNA isolation (Fig. 1) were conducted: (1) genomic DNA extraction from standard amount of biological material (5–20 sporophores) using DNeasy Plant Mini Kit (QIAGEN), (2) genomic DNA extraction from reduced amount of biological material (one sporophore) using DNeasy Plant Mini Kit (QIAGEN), (3) genomic DNA extraction from reduced amount of biological material (one sporophore) using QIAamp DNA Investigator Kit (QIAGEN) adapted to small amounts of various DNA sources (e.g., forensic samples), (4) newly designed procedure of DNA amplification (tested on a partial nuclear small-subunit rRNA gene, abbreviated 18S or SSU, hereafter referred to as SSU) from highly reduced amount of biological material (small portion of spores) using direct PCR method. The first procedure served as a well-established in literature control to ensure that the selected material was of a sufficiently good quality to conduct molecular analyses. Each procedure was applied once for each tested specimen with the exception of an additional trial to obtain SSU sequence in the procedure (4) for *Oligonema schweinitzii* with three-fold increase of spore batch collected (when the regular attempt failed).

In order to avoid cross-contamination during DNA extraction and amplification, precautions according to good laboratory practice guidelines have been applied. The site where the material was sampled was carefully cleaned with 10% solution of sodium hypochlorite (bleach) before each sampling. To minimize contamination of spores from air all sources of air blow were minimised and whenever possible closed sporocarps were sampled. In the case of the procedure (4) whenever possible closed sporocarps were punctured. As a routine, pre- and post-PCR assays were physically separated. Before and after DNA extraction, work surfaces and equipment such as plastic racks for Eppendorf tubes were carefully cleaned with 10% solution of sodium hypochlorite (bleach). During the extractions, pipette tips with filters were used. Tubes were opened and closed carefully to avoid splashing out, all reagents and tubes were always capped whenever not in use. Additionally, negative controls (‘blank’ extraction and PCR control) were always included to monitor potential contamination.

We did not standardise the amount of sampled material across species by cutting and adjusting portions of larger sporocarps, because any manipulation with open sporophores bearing easily dispersing spores may increase the risk of contamination. Instead, we selected sporophores which were fully matured, possibly untouched, fully covered with peridium in the case of species with persistent peridium or fully filled with spores in the case of species with evanescent peridium. This necessarily introduced some differences when single sporophores were used because of size variation among species. However, we found this approach the most appropriate trade-off between the standardisation of sample material and precaution against potential contamination. For procedure (1) to (3), depending on the size of sporophores and procedure applied, one or 5–20 fruiting bodies (for each species, exact number of sporophores and approximate size of one sporocarp are given in Table 2) were transferred to Eppendorf tubes under stereoscopic microscope using sterile forceps. Eppendorf tubes containing sporophores, together with sterile tungsten bead inside each tube, were frozen at −20 C for 30 min in order to make the spore walls more brittle. After freezing, the material was disrupted using Tissue Lyser II (QIAGEN) for 1 min at 30 Hz. Genomic DNA extractions were performed according to manufacturer protocol with minor modification at the beginning of the DNeasy Plant Mini Kit (QIAGEN) protocol: for the lysis of the cells, a longer incubation time (about 30 min) was applied (after Joaquina María García-Martín, personal communication). Genomic DNA was finally eluted with 100 µL in total (2 × 50 µL) of AE Buffer (DNeasy Plant Mini Kit) or with 75 µL of ATE Buffer (QIAamp DNA Investigator Kit).

The fourth procedure tested (4) used a direct PCR method. It was designed here and combined a mechanical disruption of spores using a single acid-washed glass bead (see Frommlet & Iglesias-Rodríguez, 2008), followed by performing a PCR reaction directly in the tube containing broken spores. In the first step, a single fully matured sporophore was delicately punctured by a sterile syringe needle 0.5 × 25 mm 25G ×1″ – No.18 (DB Microlance 3). Spores were taken up from inside of the sporotheca (sporocyst) to minimise contamination. Small portion of spores adhering to the tip of sterile needle was directly transferred to the 0.2 mL PCR tube containing 1 µL of Tris-EDTA buffer solution/TE-buffer (10 mM Tris-HCl, 1 mM disodium EDTA, pH 8.0) and one sterilized acid-washed glass bead (diameter 710–1,180 µm) (Sigma-Aldrich, Saint Louis, Missouri, USA). Spores suspended in the buffer were then broken by vortexing with the *Digital Vortex*-*Genie* *2 (Scientific* Industries, Bohemia, New York, USA) equipped with plastic platform with rubber cover at speed 2850 for 60 s (2 × 30 s). Until next steps, disrupted spores were kept on a PCR cooler (Eppendorf, Hamburg, Germany).

Because selected species have spores of various size and ornamentation (potentially various ability of adhesion), we manually counted number of all spores adhered to the tip of a syringe needle for five species (*Lamproderma sauteri, Licea parasitica, Oligonema schweinitzii, Perichaena corticalis* and *Stemonitopsis hyperopta*) by submersion of the needle with adhered spores in Hoyer’s medium droplet and preparing a standard permanent microscopic slide. Spores were counted under a light microscope Nikon Eclipse E-600.

In order to estimate the percentage of broken spores obtained during the procedure (4) we manually counted number of broken and unbroken spores obtained after vortexing of spores suspended in 1 µL of Tris-EDTA buffer three times for each of the abovementioned five species in 20 randomly selected field of visions at magnification of 600x under a light microscope Nikon Eclipse E-600. The percentage was counted as a share of broken spores in all observed spores (broken plus unbroken) for three counts for each species.

### PCR amplification and DNA sequencing

For all procedures, the success of extractions was evaluated by amplification of partial SSU, a genetic marker most widely employed in molecular studies dedicated to myxomycetes. Primers used for amplification were as follows: S1 and SR4Dark (Fiore-Donno et al., 2012) for dark-spored myxomycetes (*Didymium nigripes, Lepidoderma chailletii, Diderma microcarpum, Diachea leucopodia, Physarum andinum, Ph. bivalve, Leocarpus fragilis, Diacheopsis metallica, Comatricha nigra, Lamproderma sauteri, L. cucumer*) and S1 and SR4Bright (Fiore-Donno et al., 2013) for bright-spored myxomycetes (*Arcyria obvelata, Perichaena corticalis, Hemitrichia minor, Metatrichia floriformis, Oligonema schweinitzii*). Additionally, different set of primers had to be applied for other species: S1 and SU19R (Fiore-Donno et al., 2012) for *Stemonitopsis hyperopta*, 718Cri and SR11Cri (Fiore-Donno et al., 2013) for *Cribraria persoonii*, and 718FLic and SR11Lic (Fiore-Donno et al., 2013) for *Licea parasitica*.

For procedures (1) to (3), after isolation of genomic DNA, PCR amplification was conducted in a total volume of 20 µL containing 1 × REDTaq PCR Reaction Buffer (Sigma-Aldrich), 0.2 mM of each dNTPs (Sigma-Aldrich), 0.2 µM of each primer (Sigma-Aldrich), 0.1 mg/ml bovine serum albumin (BSA) (New England BioLabs, Ipswich, Massachusetts, USA), 0.05 U/µL REDTaq DNA Polymerase (Sigma-Aldrich) and 5 µL of template DNA, adjusted with ddH_2_O. PCR cycling conditions were as follows: initial denaturation for 2 min at 95 C, followed by 39 cycles (30 s at 95 C, 30 s at 52 C, 1 min at 72 C) and a final extension for 5 min at 72 C.

In the procedure (4), the PCR reaction mix was directly added to the tube containing broken spores inside (Fig. 1), using the Phire Plant Direct PCR Master Mix kit (Thermo Scientific, Waltham, Massachusetts, USA), previously used successfully in our laboratory for amplification from trace pollen samples (Suchan et al., 2019). PCR was performed in a volume of 20 µL and contained 1 × Phire Plant Direct PCR Master Mix, 0.5 µm of each primer and 1 µL of Tris-EDTA buffer with broken spores inside, adjusted with ddH_2_O (molecular biology grade, autoclaved). Volumes of reagents were established according to the manufacturer’s guidelines included in user guide. PCR cycling conditions were as follows: initial denaturation for 5 min at 98 C, followed by 40 cycles (5 s at 98 C, 5 s at 52 C, 20 s at 72 C) and a final extension for 1 min at 72 C.

All PCR products were checked with electrophoresis in 1% agarose gel. Amplicons were purified enzymatically through incubation of 4.5 µL of PCR product with the mixture of 0.52 µL Exonuclease I and 0.98 µL Shrimp Alkaline Phosphatase (SAP) (Affymetrix, Santa Clara, California, USA) for 15 min at 37 C and 15 min at 80 C. Cycle sequencing was performed in both directions (forward and reverse) with the same primers as in the PCR reaction and using BigDye Terminator v3.1 (Thermo Scientific) diluted with 5 × Sequencing Buffer (according to the product information). Cycle sequencing was performed with following conditions: 1 min at 96 C, followed by 25 cycles of 10 s at 96 C, 5 s at 50 C and 4 min at 60 C. Sequencing products were purified using EDTA/ethanol precipitation and then separated on the ABI 3500 automated DNA sequencer (Applied Biosystems, Foster City, California, USA). Obtained sequences were checked for reading errors and assembled to construct consensus sequences using CodonCode Aligner v 6.0.2 software (CodonCode Corporation, Dedham, USA). Primer sequences were trimmed off and final sequences were aligned within each species in order to check and confirm their accuracy. Sequences obtained for a given individual using the four tested procedures were of similar length (ca. 550 bp for the set of primers S1/SR4Dark, ca. 635 bp for S1/SU19R, ca. 450 bp for 718Cri/SR11Cri and ca. 340 bp for 718Lic/SR11Lic) and quality. Taxonomic affinity of obtained sequences was checked using Basic Local Alignment Search Tool (BLAST) of the NCBI GenBank database (https://www.ncbi.nlm.nih.gov/genbank/).

Sequences for each of the species collection acquired in this study using the procedure 4 were submitted to GenBank (accession numbers listed in Table 2).

## Results and Discussion

### Efficiency of the conducted DNA isolation procedures

None of the tested procedures resulted in full success in obtaining partial SSU gene (Table 2). The first procedure of genomic DNA extraction from standard amount of biological material (5–20 sporophores) using DNeasy Plant Mini Kit (QIAGEN) (approach the most often used in the literature) and the third procedure of genomic DNA extraction from reduced amount of biological material (one sporophore) using QIAamp DNA Investigator Kit (QIAGEN) failed to succeed for two species: *Didymium nigripes* and *Physarum andinum*, thus these two methods gave positive results in 17 out of 19 tested specimens (89.5%). The second procedure of genomic DNA extraction from reduced amount of biological material (one sporophore) using DNeasy Plant Mini Kit (QIAGEN) was the least efficient, giving positive results for about half of the tested specimens (10 out of 19; 52.6%). Here, we did not reduce the volume of elution buffer during the isolation procedure, compared to procedure 1 where higher amount of material was used, as lower elution volume would negatively affect the efficiency of DNA elution from membranes. To check for possible influence of lower DNA amount obtained in procedure 2, we additionally concentrated the isolates using a vacuum concentrator, reducing 10-fold the sample volume before proceeding with PCR. However, this modification did not improve the output (data not shown). This may be due to parallel concentration of potential reaction inhibitors. Our tests thus indicate that when DNeasy Plant Mini Kit is used for genomic DNA extraction, the reduction of amount of biological material considerably decreases success in obtaining results (targeted sequence). Possibly, further optimization efforts may improve to some extent the success of procedure 2 but taking into account a good yield in procedure 3, we found the latter a better alternative of isolation based on single sporophore samples.

The newly designed procedure 4 of amplification of partial SSU gene from highly reduced amount of biological material (*ca.* 600 to several thousand spores) using direct PCR method amplified 94.7% of cases and failed to yield a targeted DNA fragment in only one species, *Oligonema schweinitzii*. This method appears therefore the most efficient and outcompetes the commonly used column-based method while using a substantially smaller amount of source material (Fig. 1). Thus, it can be recommended even for specimens for which the other methods failed to give positive results and especially for collections too scanty for applying destructive sampling.

The method of mechanical disruption of spores using a single acid-washed glass bead applied in the procedure 4 has been originally used for a single algal cell (Frommlet & Iglesias-Rodríguez, 2008), thus it seems to have potential even in extremely small amount of biological material. It has also been once applied for myxomycetes (Feng & Schnittler, 2015). Because it is difficult to isolate single spore we simplified this point by application of the portion of spores adhered to the syringe needle. The number of adhered spores differed among species (*Lamproderma sauteri* – *ca.* 2,500*, Licea parasitica – ca.* 600*, Oligonema schweinitzii* – *ca.* 1,300*, Perichaena corticalis – ca.* 860 and *Stemonitopsis hyperopta –* over 15,000) most likely because of differences in spore size and ornamentation and thus ability of adhesion. The number of broken spores fraction also greatly differed among species, from 22% (SD =0.9) for *Licea parasitica,* 32% (SD = 4.0) for *Lamproderma sauteri*, 35% (SD = 9.0) for *Stemonitopsis hyperopta*, 40% (SD = 18.0) for *Oligonema schweinitzii*, up to 70% (SD = 21.0) for *Perichaena corticalis*. However, it was rather consistent across counts within the species, hence it can be considered a rather stable value characterising each species.

### Benefits and limitations of the newly proposed procedure

The commonly used procedure 1 and two other procedures (2 and 3) tested here are based on extraction of genomic DNA (Fig. 1), therefore when the extraction is efficient the isolate can be repeatedly used for amplification of several DNA fragments. The newly proposed procedure based on direct PCR method omits the multi-step process of preparing genomic DNA and leads directly to the amplification of a desired DNA fragment from broken spores suspended in a Tris-EDTA buffer (Fig. 1). Hence, in the case of a multilocus analysis there is a need of multiplied repetition of the procedure for each desired DNA fragment. However, even the multiplied repetition does not considerably increase destruction of the biological material. According to our estimation, the procedure uses up on average *ca.* 600–15,000 spores. As Tesmer & Schnittler (2007) calculated, average-size sporocarp contains 10^5^ to 10^6^ spores, so the procedure can be repeated several dozen to several hundred times before a single sporocarp is completely destroyed. Secondly, although the procedure has to be repeated, it takes much less time (about 1.5 h for 24 samples) than the procedures based on genomic DNA extraction and amplification (about 5–6 h for 24 samples—DNA Plant Mini Kit; about 20 h for 24 samples, including 16 h of incubation—QIAamp DNA Investigator Kit), because of much simpler steps and shorter PCR cycle program used (see *Material and Methods* chapter). Finally, taking into account the costs of isolation kits, the newly tested procedure is also the cheapest option. The estimated costs of reagents (excluding additional costs of materials, etc.) for the DNA isolation step together with PCR reaction calculated per one sample were the highest for procedure 3: approx. 9 Euro, medium for procedure 1 and (2): approx. 5 Euro, and the lowest for the procedure 4: approx. 0.50 Euro. The only identified disadvantage of the newly proposed procedure (as in the case of all other protocols using direct PCR method) is that by omitting the step of genomic DNA extraction it does not allow a long-term preservation of genomic DNA isolate in parallel to the herbarium specimen.

The sensitivity of the direct PCR method can appear a technically demanding issue. Because the newly designed procedure is based on exiguous fractions of specimens, it is sensitive to contamination. For instance, in the single case of failure to obtain 18S rDNA sequence from a myxomycete specimen, *Oligonema schweinitzii*, during repetition with slightly modified protocol (triple collection of spores) we obtained a sequence of another soil protist (sequence close to a representative of *Rhizaria* according to BLAST results). *Oligonema schweinitzii* is an exception among myxomycetes in its type of fructification occurrence. While other species live in soil until sporocarp formation when the plasmodium emerges above ground or other substrate and form sporocarps above ground, *O. schweinitzii* forms sporocarps within soil. Direct PCR method is effective in amplification of small quantities of DNA, therefore a special caution has also to be paid during the process of collection of spores to avoid contamination from air.

### Possibilities of application of the newly proposed procedure

Myxomycetes is one of the largest groups of Amoebozoa with ca. 1,000 species recognized and 4,000 names in use (Ronikier & Halamski, 2018). As mentioned above, *Schnittler & Mitchell (2000)* estimated that out of 866 taxa described till 2000 at the species level, 65% were known from the type locality only or were rarely reported and represented by small numbers of collections. After 2000, another *ca.* 150 have been described (our estimation based on nomenclatural database by Lado (2005–2019). It means that almost two-thirds of all described species of myxomycetes are represented by too scanty collections to be used in molecular barcoding and phylogenetic analyses using a method for DNA extraction routinely used so far in this kind of studies. It is therefore clear that the phylogeny of the whole group of myxomycetes cannot be solved using the methods used to date because it would entail detrimental consequences for scarce type collections and other very limited biological material available. According to our tests, the new procedure gives an efficient opportunity to overcome the limitation of material scarcity. We tested it on specimens belonging to four main taxonomical groups of myxomycetes (*Cribrariales, Physarales, Stemonitidales, Trichiales*) and collected up to 24 y ago. Using the same procedure, we also obtained partial SSU gene sequence for a specimen of *Didymium nivicola* Meyl. collected 38 y ago and for several specimens of various genera: *Lamproderma, Lepidoderma, Perichaena* and *Trichia* including type collections of up to 32 y old (P Janik & A Ronikier, 2019, unpublished data). Therefore, the newly designed procedure has a great potential to be used in reconstruction of reliable myxomycete phylogeny and molecular barcoding reference database including type specimens of taxa described at least during last *ca.* 40 ys but likely also beyond. Schnittler & Mitchell (2000: Fig. 1) showed that about 430 species were described between 1980 and 2000. We counted further 150 species described after 2000 so about 580 species were described during last 40 years.

Our analysis was based on the established barcode region for myxomycetes (partial SSU) and it is most directly related to building a reference database and strengthening the barcoding potential in the group. However, it is relevant not only for barcoding procedures but also to phylogenetic studies. Due to very limited availability of other DNA regions in myxomycetes, partial SSU is currently the only accessible DNA fragment to reconstruct phylogenetic relationships in this group of organisms (Leontyev et al., 2019). While no multigene phylogeny reconstruction is possible for all myxomycetes at present, it is important to note that, as shown by *Leontyev et al. (2019)*, the currently available classification based on partial SSU gene is preliminary and many relationships are unresolved due to lack of sequences for a large part of species often represented by too scanty material. In consequence, the system of classification of myxomycetes outlined by *Leontyev et al. (2019)* can be considered as a preliminary idea and not a well-founded classification to be followed because of, among others, lacking data from type species of most genera treated. Until the methods of multigene phylogeny are established in the group, the isolation/amplification procedure proposed here (procedure 4) can serve as a tool to obtain a barcode marker from scanty specimens and it can help to supplement species sampling for more comprehensive one-gene phylogeny. Importantly, other target DNA regions are also likely to be successfully sequenced using the new procedure to support trials towards multigene phylogenies although this remains subject of further studies. For example, we already succeeded – in the framework of specific projects – in amplifying EF1A region from several *Didymium nivicola* specimens and EF1A and COI genes for a few species of *Lepidoderma* including type collections (P Janik & A Ronikier, 2019, unpublished data).

Success in obtaining positive results varied among taxonomical groups. Representatives of the order *Trichiales* seem to be in general the easiest to proceed, since the partial SSU has been successfully amplified for all species and almost all procedures [one exception was *Oligonema schweinitzii* with procedure 4], even using the least efficient procedure 2. In contrast, *Physarales* appeared to be the most recalcitrant in procedures (1) to (3). Thus, the newly designed procedure may be especially helpful in improving phylogeny of *Physarales* that is known to be in urgent need of revision (Lado & Eliasson, 2017).

As the newly designed procedure is dedicated to small quantity of biological material, it can be especially recommended for checking taxonomical identity of rare species, members of morphologically unresolved species complexes, those known only from the type locality and scanty collections obtained from a moist chamber culture. Assessing the genetic affinity of taxa described based on single collections is important for binding taxonomic conclusions. For such taxa, stability of distinguishing morphological characters could not be confirmed, and genetic data may allow checking if the unique morphological traits reflect actual taxonomic distinctiveness or are rather a result of incidental malformation.

Another fundamentally important advantage of our direct PCR-based procedure is that one sporocarp can be used for morphological analysis and DNA isolation and then further stored as documentation. While a small portion of spores is collected for molecular analyses, also traditional methods used in taxonomy can be applied on the same sporocarp, i.e., permanent preparation from spores, capillitium and peridium that bear taxonomically important characters. Thus, the procedure prevents errors of analysis of two different species present in a mixed collection. Presence of two or more species in the same field collection is a frequent situation. In the case of species that represent different genera from bright- and dark-spored groups, delimitation based on macroscopic features is not problematic even with limited experience in myxomycete identification. However, when two species belong to the same genus, e.g., *Lamproderma*, the mistake is very likely, because some species cannot be differentiated based solely on macromorphological examination and delimitation based on spore size and ornamentation is necessary. This issue may likely engender classification errors and false discrepancies between molecular and morphological data in many studies based on standard procedures of DNA isolation.

Direct PCR approach has been successfully applied in many applications where either rapid procedure or amplification based on small amounts of target DNA are concerned (e.g., Wong et al., 2014; Ben-Amar, Oueslati & Mliki, 2017). It was found efficient not only in direct handling of small portions of biological material but also in analysing low-yield forensic DNA traces (Cavanaugh & Bathrick, 2018) and retrieving PCR targets from preservative medium instead of sampling the conserved specimen tissue (Shokralla, Singer & Hajibabaei, 2010). Recently, direct PCR was applied as improvement for metabarcoding of low-amount environmental samples such as pollen deposit carried by insects (Suchan et al., 2019). Here, we demonstrate that combination of adequate handling of spores aiming at efficient disruption of their microstructures, coupled with application of direct PCR on resulting suspension, can be an efficient way to circumvent technical limitations for genetic studies of myxomycete species. As it is based on widely available protocols and is simple to use, rapid and cost-effective, it can likely have a wide potential also for studies in other groups of organisms.

## Conclusions

In our analysis of DNA isolation and amplification approach we demonstrated that quantity of myxomycete material sampled for DNA analyses, the main limitation in molecular studies of the group, can be significantly reduced. In particular, the newly proposed protocol which combined micro-disruption of spores with direct PCR, which uses small portions of spores from a single sporocarp, has a potential of making even very scanty myxomycete collections accessible for phylogenetic and barcoding research, hence including in studies a large part of hitherto unavailable diversity of the group. Our test described here, followed by first applications of this method in other phylogenetic studies, indicate that it can be successfully applied to a wide taxonomical sampling, materials even dozens of years old and several DNA regions explored in the group. Future studies, especially addressing ancient collections and various target regions, will allow to further examine the potential of the method.

##  Supplemental Information

10.7717/peerj.8406/supp-1 Supplemental Information 1 ARCYRIA obvelata KRAM M-1083 18S ribosomal RNA gene partial sequence

10.7717/peerj.8406/supp-2 Supplemental Information 2 DIACHEPSIS metallica KRAM M-1456 18S ribosomal RNA gene partial sequence

10.7717/peerj.8406/supp-3 Supplemental Information 3 COMATRICHA nigra KRAM M-176 18S ribosomal RNA gene partial sequence

10.7717/peerj.8406/supp-4 Supplemental Information 4 CRIBRARIA persoonii KRAM M-1075 18S ribosomal RNA gene partial sequence

10.7717/peerj.8406/supp-5 Supplemental Information 5 DIACHEA leucopodia KRAM M-1767 18S ribosomal RNA gene partial sequence

10.7717/peerj.8406/supp-6 Supplemental Information 6 DIDYMIUM nigripes KRAM M-1771 18S ribosomal RNA gene partial sequence

10.7717/peerj.8406/supp-7 Supplemental Information 7 DIDERMA microcarpum KRAM_M-1323 18S ribosomal RNA gene partial sequence

10.7717/peerj.8406/supp-8 Supplemental Information 8 LAMPRODERMA cucumer KRAM M-133 18S ribosomal RNA gene partial sequence

10.7717/peerj.8406/supp-9 Supplemental Information 9 LEPIDODERMA chailletii KRAM M-1145 18S ribosomal RNA gene partial sequence

10.7717/peerj.8406/supp-10 Supplemental Information 10 HEMITRICHIA minor KRAM M-1638 18S ribosomal RNA gene partial sequence

10.7717/peerj.8406/supp-11 Supplemental Information 11 LEOCARPUS fragilis KRAM M-1769 18S ribosomal RNA gene partial sequence

10.7717/peerj.8406/supp-12 Supplemental Information 12 OLIGONEMA schweinitzii KRAM M-1466 18S ribosomal RNA gene partial sequence

10.7717/peerj.8406/supp-13 Supplemental Information 13 LICEA parasitica KRAM M-1599 18S ribosomal RNA gene partial sequence

10.7717/peerj.8406/supp-14 Supplemental Information 14 PERICHAENA corticalis KRAM M-1090 18S ribosomal RNA gene partial sequence

10.7717/peerj.8406/supp-15 Supplemental Information 15 PHYSARUM andinum KRAM M-1541 18S ribosomal RNA gene partial sequence

10.7717/peerj.8406/supp-16 Supplemental Information 16 METATRICHIA floriformis KRAM M-1770 18S ribosomal RNA gene partial sequence

10.7717/peerj.8406/supp-17 Supplemental Information 17 PHYSARUM bivalve KRAM M-1768 18S ribosomal RNA gene partial sequence

10.7717/peerj.8406/supp-18 Supplemental Information 18 LAMPRODERMA sauteri KRAM M-1334 18S ribosomal RNA gene partial sequence

10.7717/peerj.8406/supp-19 Supplemental Information 19 STEMONITOPSIS hyperopta KRAM M-1087 18S ribosomal RNA gene partial sequence

## Additional Information and Declarations

### Data Availability

The following information was supplied regarding data availability: 18S ribosomal RNA gene partial sequences are available at GenBank: MN104034–MN104052.
